# Assessment of Blood Pressure Control among Hypertensive Patients in Southwest Ethiopia

**DOI:** 10.1371/journal.pone.0166432

**Published:** 2016-11-23

**Authors:** Solomon Woldegebriel Asgedom, Esayas Kebede Gudina, Tigestu Alemu Desse

**Affiliations:** 1 Department of Pharmacy, Clinical Pharmacy Unit, College of Health Sciences, Mekelle University, Mekelle, Ethiopia; 2 Department of Internal Medicine, School of Medicine, Jimma University, Jimma, Ethiopia; 3 Department of Pharmacology and Clinical Pharmacy, School of Pharmacy, Addis Ababa University, Addis Ababa, Ethiopia; Fraunhofer Research Institution of Marine Biotechnology, GERMANY

## Abstract

**Background:**

The rate of blood pressure control among hypertensive patients is poor and the reasons for poor control of blood pressure remain poorly understood globally.

**Objective:**

To assess the rate of blood pressure control among adult hypertensive patients at Jimma University Specialized Hospital.

**Materials and Methods:**

We conducted a hospital based cross sectional study among adult hypertensive patients at Jimma University Specialized Hospital hypertension clinic from March 4, 2015 to April 3, 2015. Data on sociodemographic characteristics of the participants and adherence to antihypertensive medication(s) were collected from patients by face to face interview using a pretested structured questionnaire. Comorbidities, antihypertensive medication(s) and blood pressure measurements were collected retrospectively from medical records. Medication adherence was assessed using Morisky’s Medication Adherence Scale-8 score. We did the statistical analysis using chi-square test and binary logistic regression with level of α set at 0.05. Statistical significance was considered for variables with p<0.05.

**Results:**

Out of 311 participants, 286 patients were eligible and were studied. More than half, 154 (53.8%), of the participants were males. The mean age of the participants was 54.8± 12.6 years (range 26 to 94). The majority, 196 (68.53%), of the participants were taking more than one antihypertensive medication. More than one third (39.5%) of the participants were non adherent to their medication(s). The rate of blood pressure control was 50.3%. In a univariate logistic regression analyses, age ≥65 years old (P = 0.008), physical inactivity (p<0.001), chat chewing (P<0.001), adding salt to food (P<0.001), and coffee use (P<0.001) are significantly associated with uncontrolled blood pressure

**Conclusion:**

Almost half of the hypertensive patients on follow up had uncontrolled blood pressure. We recommend better health education and care of patients to improve the rate of blood pressure control at the hospital.

## Introduction

Hypertension is one of the most significant risk factors for cardiovascular diseases (CVDs). Its global burden increasing [1, 2]. It is projected to increase from approximately 1.0 billion in 2000 to 1.5 billion by 2025 [3, 4] being the leading cause of morbidity and mortality among non-communicable diseases. It is the third cause of disability adjusted life-years worldwide accounting for 13% of all deaths globally [5].

The burden of hypertension and other cardiovascular diseases is increasing in developing countries [3, 6–8]. In Africa, hypertension is both the leading risk factor for CVD and the number one cause of death. The increasing epidemics of hypertension and CVDs in Africa are important public health problems resulting in a big economic impact. This is because a significant proportion of the productive population is affected by hypertension and its complications [9]. Almost three-quarters of people with hypertension (639 million people) live in developing countries (with limited health resources) where people have a very low awareness about hypertension and BP control [10]. The prevalence of hypertension is increasing in Africa rising from 19.7% in 1990 to 30.8% in 2010 [11]. For example, in Nigeria, the prevalence of hypertension ranges from 8%-46.4% depending on the study target population [8]; in Zimbabwe, the prevalence of uncontrolled hypertension is 67.2% [12].

The majority of patients’ blood pressure remains uncontrolled in all societies [5, 13, 14] Currently, low-income and middle-income countries have the highest systolic blood pressure (SBP). In East Africa, SBP is increasing with a range of 0.8–1.6 mm Hg per decade in men and 1.0–2.7 mm Hg per decade in women while it is decreasing in Western Europe; Australia and North America [15].

Despite the availability of effective medical therapy, more than half of hypertensive patients on treatment have blood pressures above 140/90 mm Hg threshold [13, 16]. In developing countries, the high prevalence of hypertension and poor hypertension control are important factors in rising the epidemics of cardiovascular diseases [17]. Behavioral, dietary or genetic factors are responsible for uncontrolled BP [18, 19].

Uncontrolled hypertension can lead to increased incidence of complications including coronary heart disease, acute myocardial infarction, peripheral vascular disease, stroke, congestive heart failure and renal failure [19, 20]. A limited number of available data indicate that the prevalence of hypertension in Ethiopia ranges from 19.6–30% [10, 21, 22]. In Ethiopia, hypertension accounted for 1.4 percent of all deaths in 2000/01 being the seventh leading cause of death in the country for the year [18].

Recent evidence indicates that hypertension and elevated BP are increasing partly because of the increase in risk factors including smoking, obesity, harmful use of alcohol and lack of exercise [4, 23, 24]. The added burden of diseases as a consequence of uncontrolled hypertension places additional pressure on the limited health care budget in Ethiopia. In Addis Ababa, more than half, 59.9%, of the patients had uncontrolled BP [25]. A study in Southwest Ethiopia showed that only 22.4% patients had controlled hypertension [26].

There are a limited number of researches on BP control status of hypertensive in Ethiopia. The lack of adequate studies on hypertension significantly affects hypertension management and care of hypertensive patients in the country. Thus, this study aimed to assess BP control status among adult hypertensive patients at Jimma University Specialized Hospital (JUSH).

## Materials and Methods

We conducted a hospital based cross sectional study among adult hypertensive patients at Jimma University Specialized Hospital (JUSH) hypertension clinic from March 4, 2015 to April 3, 2015. Jimma University Specialized Hospital (JUSH) is both a referral and teaching hospital with 523 beds serving for, approximately, 20,000 admissions and 140, 000 outpatient visits a year. It serves for a catchment population of about 15 million people. Hypertensive patients get follow up care and antihypertensive medications at hypertension clinic every month. The clinic provides service for 1694 ambulatory hypertensive patients.

The study was approved by Jimma University Collage of Public Health and Medical Sciences Ethical Review Committee. Prior to data collection on sociodemographic characteristics and adherence of participants to antihypertensive medication(s), each participant signed a written informed consent. We conducted a face to face interview with a pretested structured questionnaire. The participants had a right to withdraw from participation. Comorbidities, antihypertensive medication(s) and blood pressure measurements were collected from medical records of patients using a pretested data abstraction format.

To maintain the validity of the data collection tool, a structured questionnaire was developed and translated to local languages (Amharic and Afan Oromo) and back translated to English. We used Morisky’s Medication Adherence Scale (MMAS-8) to assess the participants’ medication adherence.

Physical activity was assessed by asking each participant the number of minutes per day and the number of days per week a patient spent doing physical activity. The participants were classified into physically active if they reported that they were farmer or if they reported that they exercise greater than 30 minutes for greater than 5 days of the week otherwise they were classified as physically inactive. Body weight was measured to the nearest 0.1kg using a digital scale and height was measured to the nearest 0.1cm in the standing position using a portable height board. Body mass index was calculated as weight in kilogram divided by squared height (meter square).

Participants included in the study were all hypertensive patients getting follow up at JUSH hypertension clinic. Participants ≥18 years old who were on follow up for at least 12 months and whose medical records contained complete pertinent data and who were willing to participate were included. Seriously ill patients not able to complete the interview and patients with incomplete medical records such as demographics and BP were excluded. The data were collected by six clinical nurses working at Jimma University Specialized Hospital (JUSH).

Data extracted included sociodemographic characteristics, co-morbidities, adherence status of patients to antihypertensive medication(s), type of antihypertensive medication(s), duration of hypertension, and one year blood pressure measurements. The main outcome of the study was blood pressure control status.

### Statistical Analysis

We analyzed the data using SPSS Version 20.0 (Chicago, SPSS Inc.). We conducted a univariate logistic regression analyses to identify factors that are associated with uncontrolled blood pressure. Variables with P<0.05 were considered statistically significant.

### Operational definitions

Hypertension was defined as a sustained high blood pressure (SBP≥140 or DBP ≥90mmHg) or reported regular use of anti-hypertensive medication(s) [9]. Uncontrolled blood pressure was defined as systolic blood pressure of ≥140 mmHg and/or diastolic blood pressure of ≥90 mmHg [27]. Controlled blood pressure was defined as systolic blood pressure of <140 mmHg and/or diastolic blood pressure of <90 mmHg [28]. Patients were considered as adherent to their medication(s) when Morisky’s medication adherence scale (MMAS-8) score was less than 3 [29, 30] and non-adherence was considered when the patient’s MMAS-8 score was ≥3. Overweight was considered if BMI was between 25 and 29.9 kg/ m2 [31]. Obesity was defined as BMI ≥30kg/m2 or waist circumference >102 cm for men and > 88 cm for women [31].

## Results

### Baseline characteristics of the participants

In this study, a total of 311 participants were interviewed and 286 of them were eligible and studied. The response rate was 92%. As shown in table 1, one hundred fifty four (53.8%) of the participants were males. The mean age of the participants was 54.8 ± 12.6 years (range from 26–94 years). Two hundred twenty six (79.0%) of the participants were married. The most common religions were Islam, 145 (47.2%) and Orthodox Christianity, 125 (43.7%).

**Table 1:** Sociodemographic characteristics of hypertensive patients at JUSH hypertension clinic.

	Characteristics	Number (N = 286) and percentage (%)
	mean ± SD	54.8±12.6
Age in years	< 45	49(20.6)
	35–44	49(17.1)
	45–54	80(28.0)
	55–64	76(26.6)
	≥65	71(24.8)
Gender	Male	154(53.8)
	Female	132(46.2)
Marital status	Single	4(1.4)
	Married	226(79)
	Divorced	41(14.3)
	widowed	15(5.2)
Educational level	No formal education	111(38.8)
	Primary education(1-8grade)	67(32.4)
	Secondary education(9–12 grade)	46(16.1)
	Tertiary education(diploma and above)	62(21.7)
Religion	Islam	135(47.2)
	Orthodox Christian	125(43.7)
	Protestant	25(8.7)
	Others	1(0.3)
Current occupation	Civil servant	80(28.0)
	Merchant	35(12.2)
	Farmer	49(17.1)
	House wife	91(31.8)
	Retired	23(8.0)
	Jobless	8(2.8)
	mean ±SD	26.2±3 kg/m2
BMI	18.5–24.9	84(29.4)
	25–29.9	173(60.5)
	≥30	29(10.1)
Duration of hypertension in years	mean ±SD	5±4.1 years
	<5 years	165(57.7)
	≥5 years	121(42.3)

The majority, 160 (55.9%), of the participants have been taking salt with food. More than half, 158 (55.2%) of the participants were physically inactive. Nine participants (3.11%) were cigarette smokers, 48 (16.8%) were alcohol drinkers and 122 (42.7%) were chat chewers. The majority, 167 (58.4%), of the participants have been drinking coffee (Table 2). The majority, 173 (60.5%), of the participants were overweight. The mean duration of hypertension was 5 ± 4.1 years. More than half, 103 (51%), of the participants had hypertension for less than five years.

**Table 2:** Life style factors of hypertensive patients at JUSH hypertension clinic.

	Factors	Frequency (%), N = 286
Add salt to food	Yes	160(55.9)
	No	126(44.1)
Alcohol use	Yes	48(16.8)
	No	238(83.2)
Chew Chat	Yes	122(42.7)
	No	164(57.3)
	Never smoked	268(93.7)
Cigarettes	Ex-smoker	9(3.1)
	Current smoker	9(3.1)
Physical activity	Physically active	128(44.8)
	Physically inactive	158(55.2)
Drink Coffee	Yes	167(58.4)
	No	119(41.6)
Use traditional Medicine	Yes	42(14.7)
	No	244(85.3)

The majority, 167 (58.4%), of the participants had at least one co-morbidity. Seventy eight (27.2%) of the participants had diabetes, 66 (23.1%) had peripheral neuropathy, 32 (11.2%) had dyspepsia and 14 (4.9%) had hypertensive heart disease (HHD). The remaining 43 (15%) had other comorbidities (heart failure, chronic kidney disease, urinary tract infection, human immunodeficiency virus infection, ischemic heart disease, asthma, sexual dysfunction and thyrotoxicosis).

### Antihypertensive medications

Out of the 286 participants, 196 (68.53%) were prescribed with more than one antihypertensive medication. The majority, (52.1%), were on two medications and 84 (29.4%) were on mono-therapy. Seventy seven (27%) of the participants on two medications had uncontrolled BP. Fifty three (18.6%) of the participants on monotherapy had controlled blood pressure. Forty six (16.1%) participants were prescribed with triple antihypertensive medications and only one participant was on four antihypertensive medications. The number of antihypertensive medications prescribed was not associated with blood pressure control status (P = 0.094). Ninety two (32.2%) of the participants have been getting their medication for free. Dietary approach to stop hypertension (DASH) therapy was practiced by 6 (2.4%) of the participants.

Angiotensin converting enzyme inhibitors (ACEIs) and diuretics were the most commonly prescribed combination antihypertensive medication(s), 88 (30.8%). More than two third, 206 (72.0%), of the participants were prescribed with ACEIs (all enalapril) and 182 (34.7%) were prescribed with thiazide diuretics (hydrochlorothiazide) (Table 3).

**Table 3:** Antihypertensive medications prescribed at JUSH hypertension clinic.

Antihypertensive medication class	Number of patient and percentage
ACEIs Enalapril	206(72.0%)
	206(100%)
Diuretics Hydrochlorothiazide	182(63.6%)
	182(100%)
β- Blockers **Atenolol** **Metoprolol**	73(25.5%)
	72(98.6%)
	1(1.4%)
CCB **Amlodipine** **Nifedipine**	59(20.6%)
	55(93.2%)
	4(6.8%)
ARB **Losartan**	2(0.7%)
	2(100%)
Others	2(0.7%)

According to MMAS-8 score, 173(60.5%) of the participants were adherent to their antihypertensive medications (Table 4). The MMAS-8 score of the participants ranged from 0–7. No participant had MMAS-8 score of 8. Seventy four (25.9%) of the participants had a MMAS-8 score of 2 and 64 (22.3%) had a MMAS-8 score of 1. Forgetting medication(s) was the most common reason for non-adherence.

**Table 4:** Morisky’s medication adherence scale-8 score of hypertensive patients at JUSH hypertension clinic.

Adherence	MMAS-8 score	Number of participants (%)
Adherent	0	35(12.2)
	1	64(22.4)
	2	74(25.9)
Total		173(60.5)
Non-adherent	3	46(16.1)
	4	37(12.9)
	5	15(5.3)
	6	7(2.4)
	7	2(0.7)
Total		107(37.4)

### Blood pressure control and associated factors

The rate of blood pressure control in this study was 50.3%. The mean SBP was 132.13 ± 20.30 mmHg and mean DBP was 81.5±12.1 mmHg. We performed univariate analyses to identify factors associated with uncontrolled hypertension (Table 5). Age ≥65 years old (P = 0.008), physical inactivity (p<0.001), chat chewing (P<0.001), adding salt to food (P<0.001), and coffee use (P<0.001) are significantly associated with uncontrolled BP.

**Table 5:** Univariate logistic regression analyses to identify factors associated with uncontrolled blood pressure among hypertensive patients at JUSH.

Variables	Blood pressure control status		COR	95%CI	P-value
	Uncontrolled (%)	Controlled (%)			
DM					
Yes	46(59)	32(41)	1.68	1.0–2.84	0.05
No	96(46.2)	112(53.8)	1	1	1
Age in years					
< 45	26(44.1)	33(55.9)	2	1.013–3.972)	0.05
45–54	49(61.3)	37(48.7)	1	1	1
55–64	39(51.7)	43(60.6)	1.5	0.79–2.83	0.2
≥65	28(39.4)	43(60.6)	2.4	1.26–4.67	0.008
Physical activity					
Physically active	9(7.0)	119(93)	1	1	1
Physically inactive	133(84.2)	25(15.8)	7.3	31.57–156.71	<0.001
Peripheral Neuropathy					
Yes	26(39.4)	40(60.6)	0.58	0.33–1.02	0.06
No	116(52.7)	104(47.3)	1	1	1
Religion					
Orthodox	63(50.4)	62(49.6)	1.23	0.76–2.01	0.4
Protestant	18(69.2)	8(30.8)	2.73	1.11–6.71	0.29
Muslim	61(45.2)	74(54.8)	1	1	1
BMI(kg/m2)					
<18.5	35(41.7)	49(58.3)	0.7	0.42–1.19	0.2
18.5–29.9	87(50.3)	86(49.7)	1	1	1
30–35	20(69)	9(31)	2.2	0.95–5.01	0.08
Chat chewing					
Yes	95(76.6)	27(23.4)	8.7	5.08–15.11	<0.001
No	47(28.7)	117(71.3)	1	1	1
Adding salt to food					
Yes	136(85)	24(15)	33.3	44.82–286.5	<0.001
No	6(4.8)	120(95.2)	1	1	1
Drink Coffee					
Yes	135(80.8)	32(19.2)	27.5	28.69–158.76	<0.001
No	7(5.9)	112(94.1)	1	1	1
Adherence					
adherent	88(50.9)	85(49.1)	1.02	0.63–1.65	0.95
non adherent	54(50.5)	53(49.5)	1	1	1
No of anti-HTN medications					
1 medication	36(40)	54(60)	0.62	0.37–1.06	0.08
2 medications	77(51.7)	72(48.3)	1	1	1
≥3 medications	29(61.7)	18(38.3)	1.506	0.77–2.95	0.23
Duration of HTN					
<5 year	99(49)	103(51)	1	1	1
≥5 years	43(51.2)	41(48.8)	1.18	0.74–1.89	0.48
Co-morbidity					
Yes	82(49.1)	85(50.9)	0.95	0.59–1.52	0.83
No	60(50.4)	59(49.6)	1	1	1

## Discussion

In this study about half, (49.7%), of the participants had uncontrolled blood pressure. More than half, 60.5%, of the participants were adherent to their antihypertensive medications. Diabetes mellitus and peripheral neuropathy were the commonly encountered co-morbidities among hypertensive patients.

In our study more than half, 60.5%, of the study subjects were adherent to their antihypertensive medication(s). In United Arab Emirates [32], adherence to antihypertensive medication(s) was reported to be 54.4% which is lower than our finding. This may be attributed to the presence of comorbidities and advanced age of the patients which may significantly affect medication adherence. In Nigeria [33], the level of medication adherence was reported to be 33.3%. This is lower than our finding. This is because a significant number (31.4%) of the participants had depression which in turn may affect medication adherence. In our study, we excluded patients with depression and other psychiatric disorders. The level of adherence in our study is also higher than the finding in Congo (45.8%) [34]. This noted difference in adherence between ours and Congo may be explained by the difference in socio-demographic characteristics. The study participants in Congo were older than ours (mean age 63.3±9.6years vs. 54.8±12.6 years). In addition, in Congo, 53.9% participants had comorbidities; 55.9% had medication related side effects and 67.6% had stress/anxiety all of which may affect medication adherence. The level of medication adherence in our finding is lower than the Egyptians [35] which may be attributed to a difference in quality of care provided and socioeconomic differences. For example, Ethiopia is a low income country with a percapita income of $550 US dollar. The difference in the study design might also have contributed for lower level of adherence in our study. We used MMAS-8 to measure adherence and adherence was considered if the MMAS-8 is <3 while the Egyptians considered adherence if the patient took >90% of the doses in a month. Our finding is also comparable to the finding in Sudan [36] where 59.6% of patients were compliant to their medication(s) as measured with the pill count method.

The level of adherence in our finding is similar to the findings in Gondar, Northwest Ethiopia (64.6%) and Adama, Southeast Ethiopia (59.5%) [28, 37]. This might be due to similarity of socioeconomic characteristics of the participants.

We found that 27.2% of the participants had diabetes mellitus co-morbidity. This finding is in line with the finding from Brazil (29.8%), but lower than the finding from Bahrain (55.8%) [38]. This may be due to high prevalence of obesity (43.6%) in the participants enrolled in Bahrain study [38]. The low prevalence of other comorbidities in this study could be due to low availability of diagnostic materials and laboratory facilities. The level of comorbidity in our finding is also lower than the findings in Congo (53.9%) [34] which may be related to older age of participants in Congo, the difference in early identification and diagnosis of comorbidities or the difference in life style factors between ours and the Congo population.

The rate of BP control in our study was 50.3%. This is lower than the findings reported from Bahrain (66.3%) and USA (69.7%) [17,38]. The higher level of adherence in Bahrain and USA may be due to better medical and pharmaceutical care to patients, better percapita income, and better education and awareness of patients to hypertension and treatment in these countries. However, in our setup, more than 80% of the population is rural resident and the annual income is very low that may affect medication adherence.

The rate of blood pressure control is better than the findings reported by Goverwa et al (32.8%) from Zimbabwe, Lulebo et al (15.6%) from Congo, and Adebolu et al (42%) from South Africa [12,34,39]. This might be due to high prevalence of physical inactivity, bad dietary habits, obesity, the presence of comorbidities, and elderly population. For example, in South Africa, 80% of the study participants had other comorbid diseases and 66% had psychological stress. In Congo, the study participants were older than ours; 53.9% had comorbidities and 67.6% had stress or anxiety.

In Kenya, 64.7% of the renal transplant recipients attending nephrology clinics had uncontrolled hypertension (BP≥130/80 mmHg) [40] which is much higher than our finding. The difference in the study population might have contributed for this difference as the Kenyans studied renal transplant recipients only. Our finding is comparable to the finding from Egypt [35] where 53.2% participants had controlled BP while the level of adherence is higher than ours. The reason for this similarity may be because of high prevalence of comorbidities in 34.5% of the participants otherwise there is no justifiable reason for this similarity.

The rate of BP control was relatively similar to the study in Addis Ababa, Ethiopia (40.1%) and Gondar, Ethiopia (46.6%) [37, 41]. This could be due to socioeconomic similarity of the participants and almost similar level of care provided to patients.

Literatures showed that age is strongly related to systolic blood pressure and isolated systolic hypertension accounts for the majority of cases with uncontrolled BP in individuals greater than 60 years of age [42]. However, according to the joint national committee eighth meeting (JNC8) guideline, the systolic threshold for controlled hypertension is 150 mm Hg which is higher than the threshold for uncontrolled blood pressure (140 mm Hg) [2]. This could contribute to the high prevalence of uncontrolled hypertension in older age groups.

For many hypertensive patients, combination therapy is believed to achieve better BP control than monotherapy [43]. In our study, 12.6% of the participants were on monotherapy despite uncontrolled blood pressure. This is in line with the study from Zimbabwe [12]. This may be attributed to shortage of senior physicians and clinical pharmacists providing better care for ambulatory hypertensive patients to optimize appropriate combination therapy based on BP control status. The availability and cost of antihypertensive medication(s) may also be a challenge forcing the prescribers to continue with monotherapy even when it is inadequate.

In a univariate logistic regression analyses, age ≥65 years old (P = 0.008), physical inactivity (p<0.001), chat chewing (P<0.001), adding salt to food (P<0.001), and coffee use (P<0.001) are significantly associated with uncontrolled BP.

## Conclusions

Our study revealed that almost half of the participants had uncontrolled BP. More than half of the participants were adherent to their antihypertensive medications. Diabetes mellitus and peripheral neuropathy were the most commonly encountered comorbidities associated with hypertension. Age ≥65 years old (P = 0.008), physical inactivity (p<0.001), chat chewing (P<0.001), adding salt to food (P<0.001), and coffee use (P<0.001) are significantly associated with uncontrolled BP In a univariate logistic regression analyses. Based on our finding, we recommend Jimma University Specialized Hospital and Federal Ministry of Health of Ethiopia to devise strategies to provide education to hypertensive patients on dietary approach to stop hypertension (DASH) and medication adherence, develop hypertension management clinical practice guidelines and involve dedicated senior physicians and clinical pharmacists at hypertension clinic for better treatment and care of hypertensive patients.

## Supporting Information

S1 Fig Flow chart of selection of the study participants at JUSH from March 4, 2015 to April 3, 2015. (TIFF)

S2 Fig Distribution of Number of Antihypertensive Agents prescribed among adult hypertensive patients at JUSH from March 4, 2015 to April 3, 2015. (TIFF)

S1 Table Frequency of comorbidities among adult hypertensive patients at JUSH from March 4, 2015 to April 3, 2015. (DOCX)

S2 Table Frequency of Anti-hypertensive Medication Combination Regimens among Adult Hypertensive Patients at JUSH from March 4, 2015 to April 3, 2015. (DOCX)

S3 Table Univariate logistic regression analysis of factors associated with uncontrolled blood pressure among adult hypertensive patients on treatment at JUSH from March 4, 2015 to April 3, 2015. (DOCX)

S4 Table Univariate logistic regression analysis of factors associated with uncontrolled blood pressure among adult hypertensive patients on treatment at JUSH from March 4, 2015 to April 3, 2015. (DOCX)
