# Supplementary figures and images for: Assessment of Blood Pressure Control among Hypertensive Patients in Southwest Ethiopia
Source: PLoS One. 2016 Nov 23;11(11):e0166432. doi: 10.1371/journal.pone.0166432 (PMC5120816; doi:10.1371/journal.pone.0166432)

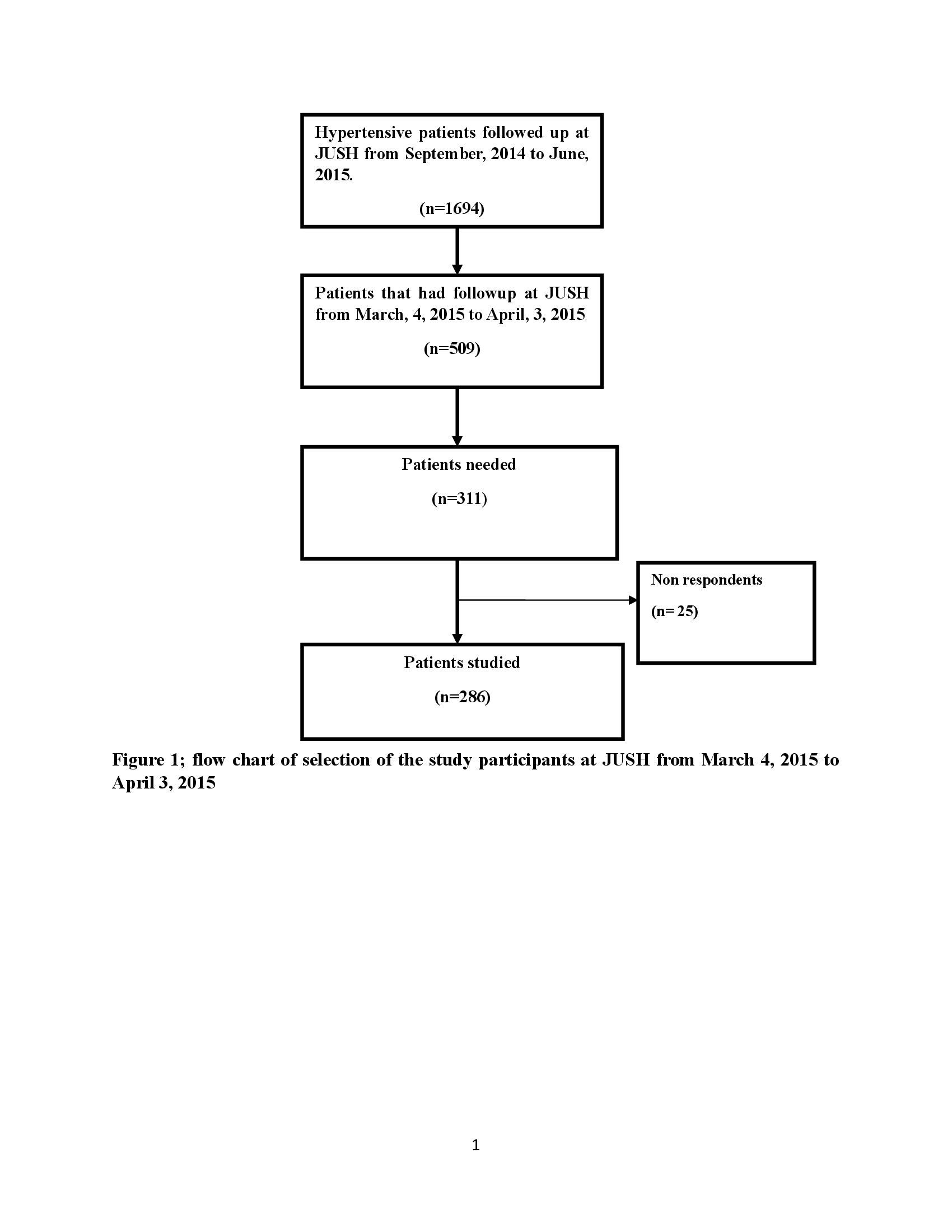

Supplement: S1 Fig — (TIFF) [file pone.0166432.s001.tiff]

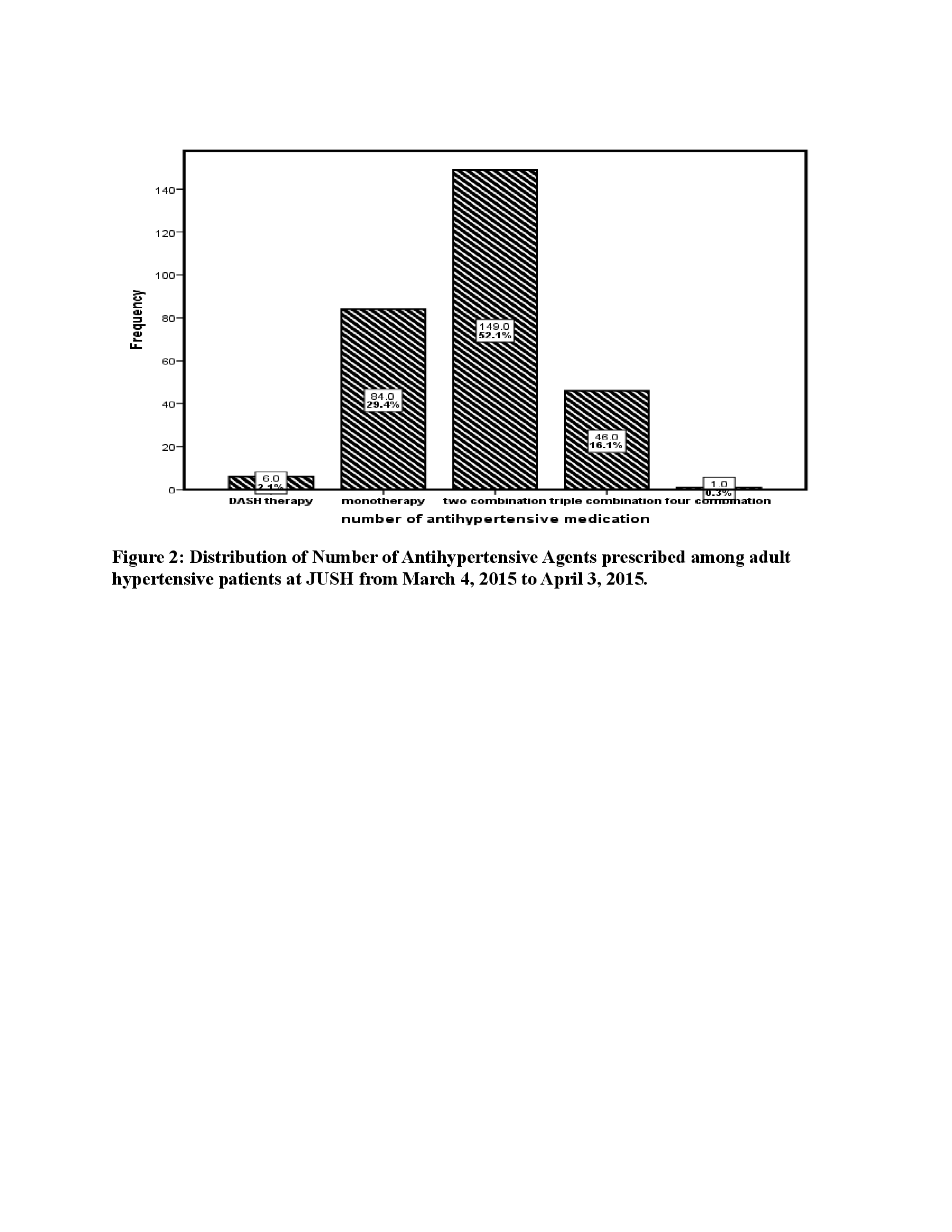

Supplement: S2 Fig — (TIFF) [file pone.0166432.s002.tiff]
