# Supplementary material for: Assessment of Blood Pressure Control among Hypertensive Patients in Southwest Ethiopia
Source: PLoS One. 2016 Nov 23;11(11):e0166432. doi: 10.1371/journal.pone.0166432 (PMC5120816; doi:10.1371/journal.pone.0166432)
Supplement: S1 Table — (DOCX) [file pone.0166432.s003.docx]

**Table 1: Frequency of comorbidities among adult hypertensive patients at JUSH from March 4, 2015 to April 3, 2015**

| Comorbidities | Frequency (%) N=286 |
| --- | --- |
| Diabetes mellitusPeripheral neuropathyDyspepsiaHHDHFCKDUTIHIV infectionIHDAsthmaSexual dysfunctionThyrotoxicosis | 78(27.2%)66(23.1%)32(11.2%)14(4.9%)8(2.8%)6(2.1%)6(2.1%)4(1.4%)4(1.4%)3(1.1%)3(1.1%)9(3.1%) |
