# Supplementary material for: Assessment of Blood Pressure Control among Hypertensive Patients in Southwest Ethiopia
Source: PLoS One. 2016 Nov 23;11(11):e0166432. doi: 10.1371/journal.pone.0166432 (PMC5120816; doi:10.1371/journal.pone.0166432)
Supplement: S2 Table — (DOCX) [file pone.0166432.s004.docx]

**Table 2: Frequency of Anti-hypertensive Medication Combination Regimens among Adult Hypertensive Patients at JUSH from March 4, 2015 to April 3, 2015.**

| Combination regimen | Frequency (%) |
| --- | --- |
| ACEI + Di | 88(30.8) |
| ACEI + BB+ Di | 23(8) |
| ACEI + BB | 15(5.2) |
| CCB+ Di | 15(5.2) |
| BB + Di | 14(4.9) |
| ACEI + CCB | 12(4.2) |
| ACEI + CCB+ Di | 10(3.5) |
| ACEI + BB + CCB | 7(2.4) |
| BB+ CCB+ Di | 6(2.1) |
| DASH therapy | 6(2.4) |
| Others | 6(2.4) |

BB: β-blockers, CCB: calcium channel blockers, ACEI: angiotensin converting enzyme inhibitors, ARB: angiotensin ІІ receptor blockers, Di: Diuretics, DASH: Dietary approach to stop hypertension, *: BB+CCB, ACEI+ BB+ CCB+ Di, Diuretic + methyldopa, ARB +CCB and ARB + BB
