# Supplementary material for: Assessment of Blood Pressure Control among Hypertensive Patients in Southwest Ethiopia
Source: PLoS One. 2016 Nov 23;11(11):e0166432. doi: 10.1371/journal.pone.0166432 (PMC5120816; doi:10.1371/journal.pone.0166432)
Supplement: S3 Table — (DOCX) [file pone.0166432.s005.docx]

**Table 3a** **univariate logistic regression analysis of factors associated with uncontrolled blood pressure among adult hypertensive patients on treatment at JUSH from March 4, 2015 to April 3, 2015.**

| Variables | Blood pressure status | | COR 95%CI | | P-value |
| --- | --- | --- | --- | --- | --- |
|  | Uncontrolled (%) | Controlled (%) |  |  |  |
| **Sex**  Male  female(1) | 80(51.9)  62(47) | 74(48.1)  70(53) | 1.221  **(1)** | 0.766-1.944  **(1)** | p=0.401  **(1)** |
| **Age**  Below 35  35-44  45-54(1)  55-64  65 & above | 3(1)  23(8)  49(17.1)  39(13.6)  43(15) | 7(2.4)  26(9.1)  31(10.8)  37(12.9)  43(15) | 0.271  0.56  **(1)**  6.67  4.12 | 0.065-1.128  0.0.273-1.149  **(1)**  0.353-1.260*  0.214-0.793* | p=0.073  p=0.114  **(1)**  p=0.045  p=0.008 |
| **Marital status**  Single/widowed  Married(**1**)  divorced | 9(47.4)  113(50)  20(48.8) | 10(52.6)  113(50)  21(51.2) | 0.900  **(1)**  0.952 | 0.352-2.298  **(1)**  0.490-1.853 | p=0.826  **(1)**  p=0.886 |
| **Occupation**  Civil servant  Merchant  Farmer  Unemployed(**1**) | 42(52.5)  19(54.3)  25(51)  56(45.9) | 38(47.5)  16(45.7)  24(49)  66(54.1) | 1.303  1.400  1.228  **(1)** | 0.740-2.292  0.658-2.976  0.632-2.384  **(1)** | p=0.359  p=0.382  p=0.545  **(1)** |
| **DM**  Yes  No(**1**) | 46(59)  96(46.2) | 32(41)  112(53.8) | 1.677  **(1)** | 0.99-2.841  **(1)** | p=0.055  **(1)** |
| **PNP**  Yes  No(**1**) | 26(39.4)  116(52.7) | 40(60.6)  104(47.3) | 0.583  **(1)** | 0.333-1.020  **(1)** | p=0.059  **(1)** |
| **Dyspepsia**  Yes  No(**1**) | 16(50)  126(49.6) | 16(50)  128(50.4) | 1.016  **(1)** | 0.487-2.119  **(1)** | p=0.967  **(1)** |
| **HHD**  Yes  No(**1**) | 7(50)  135(49.6) | 7(50)  137(50.4) | 1.015  **(1)** | 0.347-2.971  **(1)** | p=0.979  **(1)** |
