# Supplementary material for: Assessment of Blood Pressure Control among Hypertensive Patients in Southwest Ethiopia
Source: PLoS One. 2016 Nov 23;11(11):e0166432. doi: 10.1371/journal.pone.0166432 (PMC5120816; doi:10.1371/journal.pone.0166432)
Supplement: S4 Table — (DOCX) [file pone.0166432.s006.docx]

**Table 3b univariate logistic regression analysis of factors associated with uncontrolled blood pressure among adult hypertensive patients on treatment at JUSH from March 4, 2015 to April 3, 2015.**

| Variables | Blood pressure status | | COR | 95%CI | P-value |
| --- | --- | --- | --- | --- | --- |
|  | Uncontrolled (%) | Controlled (%) |  |  |  |
| **Adherence scale**  adherent  non adherent(1) | 88(50.9)  54(50.5) | 85(49.1)  53(49.5) | 1.016  **(1)** | 0.627-1.646  **(1)** | p=0.948  **(1)** |
| **No of anti-HTN medications**  ≤1 combination  2 combination(**1**)  ≥3 combination | 36(40)  77(51.7)  29(61.7) | 54(60)  72(48.3)  18(38.3) | 0.623  **(1)**  1.506 | 0.367-1.059  **(1)**  0.771-2.945 | p=0.081  **(1)**  p=0.231 |
| **Physical activity**  Physically active(**1**)  Physically inactive | 9(7.0)  133(84.2) | 119(93)  25(15.8) | **(1)**  7.342 | **(1)**  31.57-156.71***** | **(1)**  p<0.001 |
| **Alcohol intake**  Yes  No(**1**) | 24(50)  118(49.6) | 24(50)  120(50.4) | 1.017  **(1)** | 0.547-1.89  **(1)** | p=0.958  **(1)** |
| **Chat chewing**  Yes  No(**1**) | 95(76.6)  47(28.7) | 27(23.4)  117(71.3) | 8.759  **(1)** | 5.078-15.109*****  **(1)** | p<0.001  **(1)** |
| **Cigarette smoking**  Nonsmoker(1)  Ex-smoker  Current smoker | 133(49.6)  6(66.7)  3(33.3) | 135(50.4)  3(33.3)  6(66.7) | **(1)**  2.03  0.508 | **(1)**  0.497-8.286  0.124-2.071 | **(1)**  p=0.324  p=0.345 |
| **Duration of HTN**  <5 year(1)  ≥5 years | 99(49)  43(51.2) | 103(51)  41(48.8) | **(1)**  1.182 | **(1)**  0.739-1.89 | **(1)**  p=0.484 |
| **Co morbidity**  Yes  No**(1)** | 82(49.1)  60(50.4) | 85(50.9)  59(49.6) | 0.949  **(1)** | 0.593-1.518  **(1)** | p=0.826  **(1)** |
| **Cost of medicine**  Paid  Free**(1)** | 97(50)  45(48.9) | 97(50)  47(51.1) | 1.044  **(1)** | 0.636-1.716  **(1)** | p=0.864  **(1)** |

*statically significant, (1): Reference category, CI: confidence interval, COR: Crude odds ratio: DM: diabetes mellitus, PNP: peripheral neuropathy
